# Healthcare utilization and costs for patients initiating Dabigatran or Warfarin

**DOI:** 10.1186/s12955-017-0705-x

**Published:** 2017-06-21

**Authors:** Shannon L. Reynolds, Sameer R. Ghate, Richard Sheer, Pranav K. Gandhi, Chad Moretz, Cheng Wang, Stephen Sander, Mary E. Costantino, Srinivas Annavarapu, George Andrews

**Affiliations:** 1 Comprehensive Health Insights, 315 W Market St., 7th Floor, Louisville, KY 40202 USA; 2 Boehringer Ingelheim, Ridgefield, CT USA; 3 Humana Inc, Louisville, KY USA

**Keywords:** Dabigatran, Warfarin, Healthcare resource utilization, Non-valvular atrial fibrillation

## Abstract

**Background:**

Novel oral anticoagulants (NOAC) such as dabigatran, when compared to warfarin, have been shown to potentially reduce the risk of stroke in patients with non-valvular atrial fibrillation (NVAF) together with lower healthcare resource utilization (HCRU) and similar total costs. This study expands on previous work by comparing HCRU and costs for patients newly diagnosed with NVAF and newly initiated on dabigatran or warfarin, and is the first study specifically in a Medicare population.

**Methods:**

A retrospective matched-cohort study was conducted using data from administrative health care claims during the study period 01/01/2010–12/31/2012. Cox regression analyses were used to compare all-cause risk of first hospitalizations and emergency room (ER) visits. Medical, pharmacy, and total costs per-patient-per-month (PPPM) were compared between dabigatran and warfarin users.

**Results:**

A total of 1110 patients initiated on dabigatran were propensity score-matched with corresponding patients initiated on warfarin. The mean number of hospitalizations (0.92 vs. 1.13, *P* = 0.012), ER visits (1.32 vs. 1.56, *P* < 0.01), office visits (21.43 vs. 29.41; *P* < 0.01), and outpatient visits (10.86 vs. 22.02; *P* < 0.01) were lower among dabigatran compared to warfarin users. Patients initiated on dabigatran had significantly lower risk of first all-cause ER visits [hazard ratio (HR): 0.84, 95% confidence interval (CI): 0.73–0.98] compared to those initiated on warfarin. Adjusted mean pharmacy costs PPPM were significantly greater for dabigatran users ($510 vs. $250, *P* < 0.001); however, mean medical costs PPPM ($1912 vs. $1956, *P* = 0.55) and mean total costs PPPM ($2381 vs. $2183, *P* = 0.10) were not significantly different compared to warfarin users.

**Conclusions:**

Dabigatran users had significantly lower HCRU compared to warfarin users. In addition, dabigatran users had lower risk of all-cause ER visits. Despite higher pharmacy costs, the two cohorts did not differ significantly in medical or total all-cause costs.

**Electronic supplementary material:**

The online version of this article (doi:10.1186/s12955-017-0705-x) contains supplementary material, which is available to authorized users.

## Background

Atrial fibrillation (AF), the most frequently occurring type of cardiac arrhythmia, affects approximately 2.6 to 6.1 million people in the United States (US), with prevalence anticipated to increase to between 5.6 to 12 million by 2050 [1, 2]. The total annual costs for treatment of AF in the US in 2005 were estimated to be $6.0 billion [3]. The term non-valvular atrial fibrillation (NVAF) is used to describe AF which occurs without mitral valve repair, rheumatic mitral valve disease, or a prosthetic heart valve. [4] Patients with NVAF have a five-fold higher likelihood of having a stroke as compared to patients with normal sinus heart rhythm [5]. In the US, approximately 85% of AF patients are affected by NVAF, which is a substantial economic burden among those over the age of 65 years [6]. Further, strokes in patients with NVAF are often more severe and disabling as compared to strokes in patients without NVAF [7].

Clinical trial data have shown oral anticoagulation with warfarin, a vitamin K antagonist, substantially reduces the risk of stroke in patients with NVAF [8]. Warfarin was the oral anticoagulant (OAC) agent of choice and the only approved agent for AF until a few years ago [9, 10]. While many patients at high or intermediate risk for stroke are prescribed warfarin, the narrow therapeutic index, variable pharmacological effects, the need for continuous laboratory monitoring, and the slow onset of action makes maintaining a patient at a therapeutic level of effect with this drug difficult [11, 12]. Over the last few years novel oral anticoagulant (NOAC) treatment options have emerged on the market that offer the benefit of not requiring dose adjustments and international normalized ratio (INR) testing to maintain therapeutic levels within 2.0 to 3.0 [12–14]. These new therapies include direct thrombin inhibitor dabigatran, and activated factor X inhibitors rivaroxaban, apixaban, and edoxaban [15–18]. As these medications have more predictable anticoagulant effects with fewer food and drug interactions, these drugs allow for a fixed dosing regimen without the need for monitoring [19–21].

Of the NOACs, dabigatran was the first to have been approved by the Food and Drug Administration for prevention of stroke and systemic embolism in patients with NVAF in the US [22]. Several studies have previously demonstrated that dabigatran is a safe and effective alternative to warfarin for the treatment of NVAF. In the pivotal Randomized Evaluation of Long-Term Anticoagulation Therapy (RE-LY) clinical trial, when compared to adjusted-dose warfarin, dabigatran was associated with significantly lower rates of stroke, systemic embolism and intracranial hemorrhages [15]. In a retrospective database study of Medicare patients, Graham et al. [23] found that dabigatran was associated with lower risk of ischemic stroke, intracranial bleeding and death compared with warfarin and increased risk of major gastrointestinal hemorrhage associated with dabigatran compared to warfarin. Seeger et al. [24] conducted a retrospective study comparing dabigatran and warfarin using commercial health insurance databases, where the authors found a lower risk of stroke and major hemorrhage with dabigatran compared to warfarin. Pharmacoeconomic modeling based on clinical trial data have demonstrated dabigatran to be a cost-effective treatment option compared to warfarin [22, 25]. Moreover, using real-world data, patients newly diagnosed with NVAF and newly initiated on dabigatran had lower healthcare resource utilization (HCRU), higher persistence, and similar total health care costs compared to those newly initiated on warfarin [26, 27]. The objective of this study was to evaluate all-cause HCRU among patients newly diagnosed with NVAF and newly treated with dabigatran compared to newly treated with warfarin. Second, the objective was to compare all-cause healthcare costs (medical, pharmacy and total) for patients newly diagnosed with NVAF and newly initiated on treatment with dabigatran or warfarin. To the best of our knowledge, this is the first study to compare HCRU and costs among dabigatran and warfarin users using an administrative claims dataset specifically among a Medicare Advantage population in the US.

## Methods

### Study design and data source

This study aimed to compare HCRU and costs for patients newly diagnosed with NVAF and newly initiated on dabigatran or warfarin in a Medicare population. A retrospective matched-cohort study was conducted using the Humana Inc. administrative claims database. This database contains integrated medical claims, pharmacy claims, and enrollment data (Medicare or commercial plan type, member demographics, and coverage start and end dates), representing over 20 million current and former Humana members enrolled in commercial, Medicare Advantage, and prescription drug plans. The data have national coverage, with a high proportion of members from Texas, Florida, and Ohio. For this study, only the Medicare Advantage population was examined, since those who met the study criteria were predominantly Medicare members thus utilizing a majority population aged ≥65; socioeconomic status is not available in this data. Please see section on Study Population for details regarding other demographic variables that characterize this study population. The patient index date was determined using pharmacy claims and defined as the date of initiation of dabigatran or warfarin during the patient identification period from January 2011 to December 2011. Data were extracted for each patient for 12 months before the index date and up to 12 months after the index date; therefore, the full study period was from January 2010 to December 2012 (study inclusion/exclusion criteria explained in next section). The finalized protocol was approved by an independent institutional review board.

### Study population

**Fig. 1:**
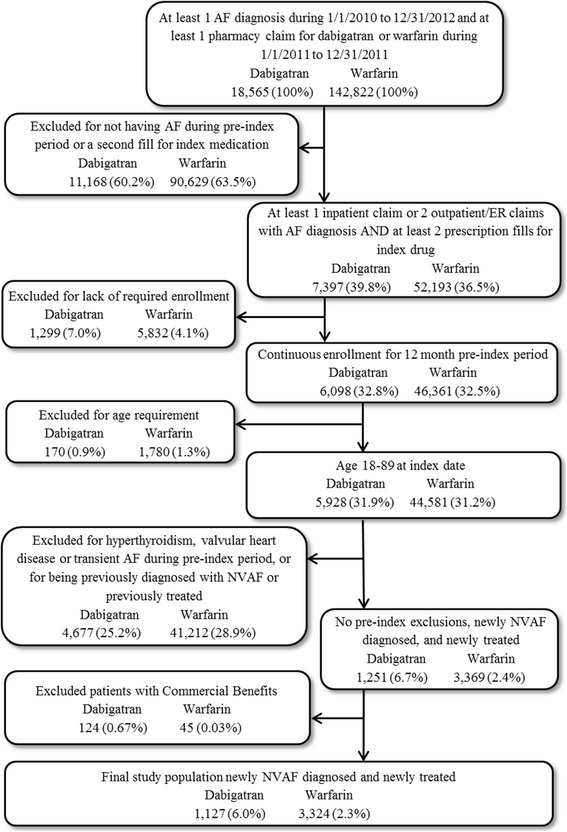
Schematic of Patient Selection

The study population consisted of patients newly diagnosed with NVAF and newly treated with dabigatran or warfarin (Fig. 1). Newly diagnosed was defined as the first AF diagnosis occurring within 3 months prior to index date and no other AF diagnosis in the >3 to 12 months before index date, while newly treated was defined as having no prescription fills for any OAC in the 12 months prior to index date. Patients were assigned to either dabigatran or warfarin cohort based on the index OAC. Inclusion criteria required patients to have the following: (1) ≥1 inpatient stay, ≥2 physician office visits, ≥2 emergency room (ER) visits, or a combination of 1 physician office visit and ER visit with an ICD-9 code of 427.31 (atrial fibrillation) on distinct service dates during the 3 month pre-index period (this definition is consistent with several previous studies conducted among NVAF patients); [26–28] (2) at least two fills of index exposure during the follow-up period (including fill on the index date); (3) 12 months of continuous enrollment prior to the index date. Criteria that excluded patients from the cohort included (1) any medical claim (inpatient stay, outpatient visit, or ER visit) with a diagnosis of hyperthyroidism or valvular heart disease during the 12 months pre-index; (2) any medical claim with a diagnosis of cardiac surgery, myocarditis, pericarditis, or pulmonary embolism (see Additional file 1 for codes) within 3 months prior to the first AF diagnosis; (3) <18 years of age or >89 years of age. A total of 1127 patients newly diagnosed with NVAF and newly initiated on dabigatran, and 3234 patients newly diagnosed with NVAF and newly initiated on warfarin were identified.

### Patient characteristics

Age, gender, race, and geographic region were reported as of the index date. The following variables derived from medical claims, were reported during the pre-index period: time from NVAF diagnosis to index date, duration of follow-up period, and comorbidities such as intracranial hemorrhage, gastrointestinal bleeding, other bleeding, ischemic stroke, heart failure, hypertension, and diabetes (see codes in Additional file 1) were assessed using medical claims for any inpatient stay, any two physician office visits or ER visits, or a combination of both (OV + ER). Clinical indexes including Deyo-Charlson [29] and Elixhauser comorbidity scores, [30] CHADS_2_ (congestive heart failure, hypertension, age ≥ 75 years, diabetes mellitus, prior stroke or transient ischemic attack), [31] CHA_2_DS_2_-VASc (congestive heart failure, hypertension, age ≥ 75 years, diabetes mellitus, prior stroke or transient ischemic attack, vascular disease, age 65–74 years, sex) [32] and HEMORR_2_HAGES [26] [hepatic or renal disease, ethanol abuse, malignancy, older age (>75), reduced platelet count or function, rebleeding risk, hypertension, anemia, genetic factors, excessive fall risk, stroke] risk scores were calculated during the pre-index period.

### Propensity score matching

Propensity score matching (PSM) based on 1:1 nearest neighbor method with a caliper width of 0.2 standard deviations (SDs) of the estimated logit was used to match dabigatran and warfarin cohorts. Propensity score matching was conducted using logistic regression with predictor variables that were known to or could greatly impact the propensity to prescribe one medication over another based on information from the research literature [26] and expert clinical and research opinion (age, gender, geographic region, Deyo-Charlson comorbidity score, HEMORR2HAGES score, intracranial hemorrhage, gastrointestinal bleed, other bleed, ischemic stroke, heart failure, hypertension, and diabetes). In addition, bivariate analyses were conducted to identify differences at baseline among the study cohorts.

### Outcomes

Patients were followed for up to 12 months post-index to evaluate HCRU and costs. End of follow-up was defined as disenrollment from the health plan, death, discontinuation (failure to refill within 30 days from the date of the previous prescription) or switch from the index medication, or end of study period (December 2012), whichever occurred first. All-cause HCRU based on primary diagnosis (see Additional file 1 for codes) was examined by place of service (inpatient hospital, ER visit, physician office visit, and other outpatient visits). Costs were estimated based on total payment to the provider, including both the plan cost and patient share. All-cause costs were presented as total costs (sum of medical and pharmacy costs), medical costs, and pharmacy costs; all costs were adjusted to 2012 dollars to account for inflation.

### Statistical analyses

Demographic and baseline characteristics were compared among dabigatran and warfarin cohorts both pre- and post-PSM. Chi-square tests and t-tests or Wilcoxon rank-sum tests were conducted for categorical and continuous variables, respectively.

The number and rates of HCRU were reported as per-patient-per-year (PPPY, eg, PPPY rate was the number of hospitalizations/visits divided by the total patient years). All-cause HCRU was calculated as the count of distinct hospitalizations, ER visits, physician office visits, or outpatient visits. Since patients could have more than one hospitalization or visit, this number was greater than the count of patients. Patient years was calculated as the number of days a patient contributed to the denominator (ie, days from index prescription until discontinuation of therapy, switching of therapy, disenrollment, death, or end of study period) divided by 365. Descriptive statistics [N, mean, SD, median, and interquartile range (IQR)] were used to report all-cause HCRU and rates were compared using a Wilcoxon rank-sum test. Additionally, Cox regression analyses were used to investigate the association between anticoagulation treatment (dabigatran or warfarin) and time to first all-cause inpatient hospitalization and ER visit, adjusting for covariates. Hazard ratios (HRs) with 95% confidence intervals (CI) and *P* values were reported.

Total (medical + pharmacy) costs, medical costs, and pharmacy costs were reported as per-patient-per-month (PPPM). Medical costs were presented separately by place of service and by event. Due to the non-normal distribution and skewed nature of cost data, unadjusted and adjusted generalized linear models (GLM) with a log link were conducted to assess the relationship between index treatment (dabigatran or warfarin) and (1) total costs, (2) medical costs, and (3) pharmacy costs. All regression models were adjusted for age, gender, race, CHADS_2_ score and HEMORR_2_HAGES score. These variables were chosen based on statistical significance or clinical relevance. The a priori alpha level for all inferential analyses was set at 0.05.

## Results

### Pre-index characteristics

Post-PSM, 1110 patients were retained in each cohort. Among all matched patients across the cohorts, the mean age was 75 years and 51% were male. With the exception of race/ethnicity (White: 93% dabigatran vs. 90% warfarin; *P* = 0.04), all other demographic and clinical characteristics were similar between the two cohorts (Table 1).

**Table 1:** Demographic and clinical characteristics of patients with NVAF treated with dabigatran or warfarin

	Pre-PSM			Post-PSM		
Measure	Dabigatran	Warfarin	*P* value	Dabigatran	Warfarin	*P* value
N	1127	3234		1110	1110	
Age, years						
Mean ± SD	75.0 ± 6.9	75.3 ± 7.4	0.3414	75.1 ± 6.9	75.0 ± 7.0	0.5682
Median, [Min – Max]	75 [42–89]	76 [40–89]		75 [42–89]	75 [46–89]	
Male, n (%)	579 (51.4)	1663 (51.4)	0.9783	569 (51.3)	574 (51.7)	0.8318
Race/Ethnicity, n (%)						
White	1043 (92.5)	2908 (89.9)	0.0093	1028 (92.6)	1000 (90.1)	0.0345
Other	84 (7.5)	326 (10.1)		82 (7.4)	110 (9.9)	
Geographic Region, n (%)						
Northeast	27 (2.4)	82 (2.5)	<0.0001	27 (2.4)	23 (2.1)	0.4615
Midwest	254 (22.5)	1004 (31.0)		254 (22.9)	254 (22.9)	
South	764 (67.8)	1868 (57.8)		747 (67.3)	768 (69.2)	
West	82 (7.3)	280 (8.7)		82 (7.4)	65 (5.9)	
Baseline Deyo-Charlson Comorbidity Index (DCI) score	2.1 ± 2.1	2.9 ± 2.5	<0.0001	2.1 ± 2.1	2.1 ± 2.0	0.5809
Baseline Elixhauser Comorbidity index (ECI) score	4.4 ± 2.5	5.5 ± 2.7	<0.0001	4.4 ± 2.5	4.5 ± 2.4	0.3789
Baseline stroke risk						
CHADS2 stroke risk score	2.3 ± 1.2	2.6 ± 1.2	<0.0001	2.3 ± 1.2	2.3 ± 1.2	0.7783
CHA2DS2-VASc stroke risk score	4.0 ± 1.5	4.3 ± 1.5	<0.0001	4.0 ± 1.5	4.0 ± 1.5	0.8061
Baseline bleeding risk						0.1780
HEMORR2HAGES score	2.4 ± 1.5	2.9 ± 1.7	<0.0001	2.4 ± 1.5	2.4 ± 1.4	
Time from first AF to index (days)	20 ± 22	19 ± 22	0.0282	20 ± 22	18 ± 22	0.0559
Duration of follow-up (days)						
Mean ± SD	346 ± 62	339 ± 74	0.0010	346 ± 62	343 ± 68	0.3329
Median [Range]	365 [42–365]	365 [9–365]		365 [42–365]	365 [11–365]	
Total HCRU pre-index, mean visits per patient [SD]						
Hospitalization	0.8 [0.7]	1.1 [0.9]	<.0001	0.8 [0.7]	0.9 [0.7]	0.0183
ER visit	0.7 [1.2]	0.8 [1.5]	0.3055	0.7 [1.3]	0.7 [1.1]	0.0603
Physician office visit	12.4 [9.6]	12.3 [10.3]	0.7100	12.4 [9.7]	11.6 [9.2]	0.0293
Outpatient visit	5.7 [6.8]	7.3 [14.0]	<.0001	5.7 [6.8]	6.1 [11.6]	0.3758

*SD* standard deviation, *HCRU* Healthcare Resource Utilization, *ER* emergency room

### Healthcare resource utilization

Total HCRU (PPPY) based on place of service among dabigatran and warfarin cohorts is presented in Table 2. Based on the study findings, there were significantly lower mean annualized HCRU rates for patients initiated on dabigatran than for patients initiated on warfarin in all places of service (Table 2). For instance, the mean number of PPPY all-cause hospitalizations was 0.92 vs. 1.13 (*P* = 0.01), ER visits were 1.32 vs. 1.56 (*P* < 0.01), physician office visits were 21.43 vs. 29.41 (*P* < 0.01), and outpatient visits were 10.86 vs. 22.02 (*P* < 0.01) in the dabigatran compared to warfarin cohorts, respectively.

**Table 2:** Total healthcare resource utilization in patients with NVAF treated with dabigatran or warfarin

Outcome	Dabigatran	Warfarin	*P* value^b^
N	1110	1110	
Hospitalization			
Number of patients hospitalized, n (%)	284 (25.6)	332 (29.9)	
All cause hospitalizations, n	381	475	
Mean PPPY^a^ [SD] (95% CI)	0.92 [3.10] (0.74, 1.11)	1.13 [2.90] (0.96, 1.30)	0.0124
Median PPPY^a^ (25p, 75p)	0.0 (0.0, 1.0)	0.0 (0.0, 1.0)	
ER visits			
Number of patients with an ER visit, n (%)	309 (27.8)	378 (34.1)	
All cause ER visits, n	563	780	
Mean PPPY^a^ [SD] (95% CI)	1.32 [4.04] (1.08, 1.56)	1.56 [3.74] (1.34, 1.78)	0.0011
Median PPPY^a^ (25p, 75p)	0.0 (0.0, 1.0)	0.0 (0.0, 1.6)	
Physician office visits			
Number of patients with a physician office visit, n (%)	1068 (96.2)	1084 (97.7)	
All cause physician office visits (n)	11,571	18,765	
Mean PPPY^a^ [SD] (95% CI)	21.43 [17.98] (20.37, 22.49)	29.41 [18.68] (28.31, 30.51)	< 0.0001
Median PPPY^a^ (25p, 75p)	17.0 (10.2, 27.3)	26.4 (16.5, 39.0)	
Outpatient visits			
Number of patients with an outpatient visit, n (%)	945 (85.1)	1040 (93.7)	
All cause outpatient visits (n)	5563	13,029	
Mean PPPY^a^ [SD] (95% CI)	10.86 [15.44] (9.95, 11.77)	22.02 [23.37] (20.64, 23.39)	< 0.0001
Median PPPY^a^ (25p, 75p)	6.6 (3.0, 12.4)	16.6 (6.7, 29.0)	

*PPPY* per-patient-per-year, *SD* standard deviation; *CI* confidence interval, *p* percentile, *ER* emergency room

^a^PPPY was calculated at the patient level (number of HCRU divided by follow-up months), and summary statistics were derived from this measure. Patients with no HCRU for a particular place of service or event are included in the calculation of PPPY since they contributed time to the denominator

^b^ *P* values calculated using a Wilcoxon rank-sum test

Adjusting for covariates, dabigatran was associated with a significantly lower risk of all-cause first ER visit [HR = 0.84 (95% CI 0.73, 0.98)]. The risk of all-cause first inpatient hospitalization was comparable between the dabigatran and warfarin cohorts [HR = 0.90 (95% CI 0.77, 1.06)].

### Healthcare costs

Using GLM to calculate predicted means, the unadjusted and adjusted PPPM mean total costs, total pharmacy costs, total medical costs, and medical costs associated with place of service are presented in Table 3. The results demonstrated that only pharmacy costs were significantly different across the two treatment cohorts. However, the adjusted mean total costs and medical costs were not significantly different between the dabigatran and warfarin cohorts. The adjusted cost results for medical costs based on place of service were also not significantly different for any of the four types of place of service. Within all-cause medical costs based on place of service, hospitalization-related medical costs accounted for a majority of these costs. Using both unadjusted and adjusted GLM, similar findings were reported between the two treatment cohorts (Table 3).

**Table 3:** Healthcare costs per person per month^a^ in patients with NVAF treated with dabigatran or warfarin

Outcome	Dabigatran	Warfarin	*P* value^b^	
Total Cost, mean (95% CI)				
Unadjusted GLM Mean	$2362 ($2213, $2521)	$2204 ($2065, $2352)	0.1398	
Adjusted GLM Mean^d^	$2381 ($2106, $2700)	$2183 ($1929, $2481)	0.0967	
Total Pharmacy Cost, mean (95% CI)				
Unadjusted GLM Mean	$509 ($488, $531)	$251 ($240, $262)	<.0001	
Adjusted GLM Mean^d^	$510 ($470, $555)	$250 ($230, $273)	<.0001	
Total Medical Cost, mean (95% CI)				
Unadjusted GLM Mean	$1903 ($1757, $2060)	$1973 ($1823, $2135)	0.5276	
Adjusted GLM Mean^d^	$1912 ($1646, $2231)	$1956 ($1683, $2286)	0.5500	
Hospital-related All-Cause Medical Cost, mean (95% CI)				
Unadjusted GLM Mean	$1534 ($1283, $1833)	$1596 ($1338, $1903)	0.7552	
Adjusted GLM Mean^d^	$1094 ($821, $1480)	$1137 ($851, $1539)	0.6607	
ER related All-Cause Medical Cost, mean (95% CI)				
Unadjusted GLM Mean	$73 ($65, $83)	$82 ($73, $92)	0.2036	
Adjusted GLM Mean^d^	$72 ($58, $91)	$83 ($66, $104)	0.0967	
Physician office visits related All-Cause Medical Cost, mean (95% CI)				
Unadjusted GLM Mean	$285 ($269, $303)	$287 ($270, $305)	0.8929	
Adjusted GLM Mean^d^	$282 ($252, $317)	$289 ($258, $326)	0.3792	
Outpatient visits related All-Cause Medical Cost, mean (95% CI)				
Unadjusted GLM Mean	$399 ($361, $441)	$360 ($325, $398)	0.1554	
Adjusted GLM Mean^d^	$396 ($327, $482)	$362 ($300, $445)	0.3120	

*CI* confidence interval, *GLM* generalized linear mean

^a^Per-patient-per-month (PPPM) was calculated at the patient level (total costs divided by follow-up months), and summary statistics were derived from this measure

^b^ *P* values derived using a generalized linear model (GLM) with log link function and gamma distribution; Service location specific costs were increased by a constant of $1 to account for zero cost in the log cost model

^d^adjusted for age, gender, race, CHADS2 score and HEMORR2HAGES score

## Discussion

Our study findings provide important insights into real-world HCRU and cost data of Medicare Advantage patients newly diagnosed with NVAF and newly treated with dabigatran compared to warfarin. We found that dabigatran users had significantly lower mean all-cause HCRU compared to patients newly initiated on treatment with warfarin. In particular, dabigatran users had significantly fewer physician office visits, outpatient visits, ER visits, and hospitalizations than warfarin users. The dabigatran users incurred a mean of 27% fewer office and 51% fewer outpatient visits. Lower office and outpatient visits for patients taking dabigatran may be due to the decreased need for anticoagulation monitoring among these patients [33]. We also found 15% fewer mean ER visits and 18% fewer hospitalizations among dabigatran users compared to warfarin users. Greater utilization of ER visits and hospitalizations may reflect poorer clinical outcomes due to suboptimal control of the INR among warfarin users. [34] Our study findings demonstrate that patients initiated on dabigatran had a lower risk of all-cause ER visits when compared to patients initiated on warfarin. Risks of all-cause incident hospitalizations were similar between the dabigatran and warfarin cohorts.

Not surprisingly, the mean pharmacy costs were significantly higher among dabigatran users compared to warfarin users. However, the mean total costs were similar among the two cohorts which can be attributed to lower medical costs (although not significantly different) among patients treated with dabigatran compared to those treated with warfarin that offset the higher pharmacy costs. Our results are comparable to recently published studies whose study methodology closely resembled the approach we used to identify patients newly diagnosed with NVAF and newly treated with dabigatran or warfarin [26, 27]. For instance, a study by Francis et al. [26] found significantly lower medical costs among dabigatran users compared to warfarin users possibly due to significantly fewer hospitalizations. Although the authors reported higher pharmacy costs for dabigatran users, overall healthcare costs were significantly lower compared to warfarin users. In comparison, although our study found significantly higher pharmacy costs among dabigatran users, the total costs and medical costs were similar to warfarin users. The difference in findings may be due to differences in pre-index patient characteristics between the two studies. Dabigatran users in our study were older (75 vs 72 years). Second, the US Department of Defense dataset utilized by Francis et al. [26] was geographically diverse with 14% of the beneficiaries on active duty; and third, the health systems across the two datasets are different that may explain the discrepant findings. Our results are consistent with the study by Bancroft et al. [27] where the authors found significantly lower all-cause HCRU (ie, ER visits, physician office visits and outpatient visits) among dabigatran users compared to warfarin users. Furthermore, the authors reported significantly higher pharmacy costs, and similar total and medical costs among dabigatran users compared to warfarin users. Of note, this growing evidence of real-world data, especially in a Medicare Advantage population, highlights significantly lower HCRU among dabigatran users compared to warfarin users. In addition, although the pharmacy costs are significantly higher, medical costs are lower and total costs are comparable to warfarin users. One of the plausible explanations may be due to the use of a Medicare Advantage population in this study and/or differences of cost-to-charge ratio among providers and higher patient sharing of expenses that could have led to comparable medical costs and total costs among dabigatran and warfarin users in this study. Future studies should examine HCRU and costs among dabigatran and warfarin users in a larger sample size and a wider geographic distribution of Medicare Advantage patients in the US.

### Limitations

Similar to other retrospective database studies, this study is subject to coding errors of omission and commission, incomplete claims, and unreliable clinical coding. As claims data were used, patients may have been previously diagnosed while not enrolled with Humana, information for which was not available for this study. It is plausible that patients treated with warfarin may or may not have been properly anticoagulated during the follow-up period. However, INR values were not available for all patients prescribed warfarin, which was not accounted for in this study.

For sufficient OAC exposure among the final study cohort, a minimum of two prescription claims of corresponding index OAC were required, which could introduce a time loop/immortal time bias requiring patients to survive in order to fill the second prescription which may result in bias in either direction. In order to evaluate the potential selection bias of requiring two fills, the baseline characteristics of patients with only one fill of index exposure for dabigatran or warfarin were assessed and were found to be similar to the population of patients with two or more fills.

## Conclusions

Among patients newly diagnosed with NVAF and newly treated, patients initiated on dabigatran experienced significantly fewer hospitalizations, ER, physician office, and outpatient visits compared to those initiated on warfarin. Although pharmacy costs were significantly higher in the dabigatran cohort, there was no significant difference in mean total costs and medical costs among dabigatran and warfarin cohorts.

## Additional file

Additional file 1: Coding algorithms used for study exclusion criteria, driver-specific events and construction of comorbidity score measures. ICD-9-CM coding algorithms used for study exclusion criteria, driver-specific events and construction of comorbidity score measures. (DOCX 21 kb)

## Abbreviations

AF: Atrial fibrillation
CI: Confidence interval
ER: Emergency room
GLM: Generalized linear model
HCRU: Healthcare resource utilization
HR: Hazard ratio
INR: International normalized ratio
IQR: Interquartile range
MI: Myocardial infarction
NOAC: Novel oral anticoagulant
NVAF: Non-valvular atrial fibrillation
OAC: Oral anticoagulant
PPPM: Per patient per month
PPPY: Per patient per year
PSM: Propensity score matching
SD: Standard deviation
US: United States
