# Supplementary material for: Healthcare utilization and costs for patients initiating Dabigatran or Warfarin
Source: Health Qual Life Outcomes. 2017 Jun 21;15:128. doi: 10.1186/s12955-017-0705-x (PMC5480105; doi:10.1186/s12955-017-0705-x)
Supplement: Additional file 1: — Coding algorithms used for study exclusion criteria, driver-specific events and construction of comorbidity score measures. ICD-9-CM coding algorithms used for study exclusion criteria, driver-specific events and construction of comorbidity score measures. (DOCX 21 kb) [file 12955_2017_705_MOESM1_ESM.docx]

**Additional file 1**

**Coding Algorithms Used For Study Exclusion Criteria, Driver-Specific Events and Construction of
Comorbidity Score Measures**

| **Variable** | **Codes** | **Code Type** |
| --- | --- | --- |
| **Codes Used to Define Exclusion Criteria** | | |
| Cardiac Surgery | 00.5x  35.xx  36.xx  37.xx | ICD-9 Procedure |
| Pericarditis | 391.x  393  420.x  423.2  036.41  074.21  093.81  098.83 | ICD-9 Diagnosis |
| Myocarditis | 391.2  422.xx  074.23  398.0  429.0  032.82  036.43  093.82  130.3 | ICD-9 Diagnosis |
| Pulmonary Embolism | 415.1x | ICD-9 Diagnosis |
| Hyperthyroidism | 242.x | ICD-9 Diagnosis |
| Valvular heart disease | 394.0x  394.2  396.0  396.1  35.20  35.22  35.24  35.26  35.28 | ICD-9 Diagnosis |
|  | 33999  0257T  0258T  0259T  33405  33425  33426  33427  33430  0262T  33475  33460  33463  33464  33465  33999  0257T  0258T  0259T  33405  33425  33426  33427  33430  0262T  33475  33460  33463  33464  33465 | CPT |
| **Codes Used to Define Driver-Specific Events** | | |
| **Bleed Related (a, b and c)** | | |
| 1. Intracranial Hemorrhage   (Regardless of position) | 430  431  432  852.0x  852.2x  852.4x  853.0 | ICD-9 Diagnosis |
| b. Gastrointestinal (Primary) | 455.2,  455.5  455.8  456.0  456.20  459.0  530.7  530.82  531.0  531.2  531.4  531.6  532.0  532.2  532.4  532.6  533.0  533.2  533.4  533.6  534.0  534.2  534.4  534.6  535.01  535.11  535.21  535.31  535.41  535.51  535.61  537.83  562.02  562.03  562.12  562.13  568.81  569.3  569.85  578 | ICD-9 Diagnosis |
| c. Other Bleeding (Primary) | 599.7  719.1x  786.3  423.0  593.81  784.7  784.8 | ICD-9 Diagnosis |
| MI-Related | 410.xx | ICD-9 Diagnosis |
| Stroke-Related | 433.x1, 434.x1, 436.x | ICD-9 Diagnosis |
| **Codes Used to Define Comorbid Conditions of Interest and Disease Scores** | | |
| Congestive Heart Failure | 398.91, 402.11, 402.91, 404.11, 404.13, 404.91, 404.93, 428.0x-428.9x | ICD-9 Diagnosis |
| Cardiac arrhythmias | 426.10, 426.11, 426.13, 426.2-426.53, 426.6x-426.89, 427.0x, 427.2x, 427.31, 427.60,427.9x, 785.0x, V45.0x, V53.3x | ICD-9 Diagnosis |
| Valvular disease | 093.20-093.24, 394.0x-397.1x, 424.0x-424.91,746.3x-746.6,V42.2,V43.3x | ICD-9 Diagnosis |
| Pulmonary Circulation disorders | 416.0x-416.9x, 417.9x | ICD-9 Diagnosis |
| Peripheral vascular disease | 440.0x-440.9x, 441.2x, 441.4x, 441.7x, 441.9x, 443.1x-443.9x, 447.1x,557.1x, 557.9x, V43.4x | ICD-9 Diagnosis |
| Hypertension, uncomplicated | 401.1x, 401.9x | ICD-9 Diagnosis |
| Hypertension, complicated | 402.10, 402.90, 404.10, 404.90, 405.11, 405.19, 405.91, 405.99 | ICD-9 Diagnosis |
| Paralysis | 342.0x-342.12, 342.9x-344.9x | ICD-9 Diagnosis |
| Other neurological disorders | 331.9x, 332.0x, 333.4x,333.5x,334.0x-335.9x,340.xx, 341.1x-341.9x,345.00-345.11, 345.40-345.51, 345.80-345.91, 348.1x,348.3x, 780.3x, 784.3x | ICD-9 Diagnosis |
| Chronic pulmonary disease | 490.xx-492.8x, 493.00-493.91, 494.xx, 495.0x-505.xx, 506.4x | ICD-9 Diagnosis |
| Diabetes, uncomplicated | 250.00-250.33 | ICD-9 Diagnosis |
| Diabetes, complicated | 250.40-250.73, 250.90-250.93 | ICD-9 Diagnosis |
| Hypothyroidism | 243.xx-244.2x, 244.8x, 244.9x | ICD-9 Diagnosis |
| Renal failure | 403.11, 403.91, 404.12, 404.92, 585.xx, 586.xx, V42.0x,V45.1x,V56.0x,V56.8x | ICD-9 Diagnosis |
| Liver disease | 070.32, 070.33, 070.54, 456.0x, 456.1x, 456.20, 456.21 571.0x, 571.2x, 571.3x, 571.40-571.49, 571.5, 571.6x, 571.8x, 571.9x,572.3x,572.8x, V42.7x | ICD-9 Diagnosis |
| Peptic ulcer disease excluding bleeding | 531.70, 531.90, 532.70, 532.90, 533.70, 533.90,534.70,534.90, V12.71 | ICD-9 Diagnosis |
| Acquired immune deficiency syndrome(AIDS) | 042.xx-044.9x | ICD-9 Diagnosis |
| Lymphoma | 200.00-202.38, 202.50-203.01,203.8-203.81, 238.6x, 273.3x,V10.71,V10.72,V10.79 | ICD-9 Diagnosis |
| Metastatic cancer | 196.0x-199.1x | ICD-9 Diagnosis |
| Solid tumor without metastasis | 140.0x-172.9x,174.0x-175.9x,179.xx-195.8x, V10.00-V10.9x | ICD-9 Diagnosis |
| Rheumatoid arthritis/collagen vascular diseases | 701.0x, 710.0x-710.9x, 714.0x-714.9x, 720.0x-720.9x, 725.xx | ICD-9 Diagnosis |
| Coagulopathy | 286.0x-286.9x, 287.1x, 287.3x-287.5x | ICD-9 Diagnosis |
| Obesity | 278.0x | ICD-9 Diagnosis |
| Weight loss | 260.xx-263.9x | ICD-9 Diagnosis |
| Fluid and electrolyte disorders | 276.0x-276.9x | ICD-9 Diagnosis |
| Iron deficiency anemia secondary to blood loss (chronic) | 280.0x | ICD-9 Diagnosis |
| Deficiency anemias | 280.1x-281.9x, 285.9x | ICD-9 Diagnosis |
| Alcohol abuse | 291.1x, 291.2x, 291.5x, 291.8x, 291.9x, 303.90-303.93,305.00-305.03, V113 | ICD-9 Diagnosis |
| Drug abuse | 292.0x, 292.82-292.89,292.9x,304.00-304.93, 305.20-305.93 | ICD-9 Diagnosis |
| Psychoses | 295.00-298.9x, 299.10-299.11 | ICD-9 Diagnosis |
| Depression | 300.4x, 301.12, 309.0x, 309.1, 311.xx | ICD-9 Diagnosis |
